# On the Use of Large Doses of Opium in the Treatment of Fever, Inflammation, and Dysentery

**Published:** 1852-01

**Authors:** Anson G. Henry

**Affiliations:** Springfield, Illinois


### WESTERN JOURNAL

OF

### MEDICINE AND SURGERY.

JANUARY, 1852.

---

### Art. I.—*On the Use of Large Doses of Opium in the Treatment of Fever, Inflammation, and Dysentery.* By Anson G. Henry, M. D., of Springfield, Illinois.

I propose in as brief a manner as possible, in the following communication, to call the attention of the profession to the use of large doses of opium, (and by large doses, I mean from four to six grains,) *as the main curative agent, in the first or forming stage, of Fever, Inflammation, and Dysentery.*

As early as the year 1837, I made an effort under the patronage of my late lamented friend, Doctor John P. Harrison, of the Ohio Medical College, to call attention to the safety and efficiency of large doses of opium in fever; and with that view, reported for publication in the Western Medical Journal, at Cincinnati, several cases of fever which I had treated, with very happy results, with four grain doses of opium. Dr. Harrison discouraged their publication, on the ground that it would not be prudent or politic to put at hazard my medical reputation by the promulgation of doctrines so directly at war with the well-settled theories and practice of the profession. This rebuff from a very learned and highly esteemed medical friend, not only induced me to doubt the correctness of my conclusions, although founded at the bed side of my patients, but rendered me very timid in the use of the large dose, until all the usual remedies had proved unavailing. But its use under these most unfavorable circumstances, so frequently rescued my patients from a state of extreme peril, and placed them in one of safety, that I could not avoid the conviction that it was a remedy of very great power, and I began to venture upon its use the moment dangerous symptoms showed themselves. In 1840, I commenced its use as my main remedy, having become fully satisfied that no danger was to be apprehended from an over dose of opium, or from congestion of the brain. I found that in precisely similar cases, where the dose of the books had evidently aggravated the cerebral symptoms, increased dryness of skin, and failed to relieve permanently the patient, a large dose seldom failed to produce full and permanent relief. I became satisfied that the doctrine so generally, and assiduously inculcated and practiced upon by the profession, viz: that the use of opium was to be carefully avoided, in all cases of fever where there was determination to the brain, did not apply to the large dose, however well it might apply to the largest dose recommended by medical writers, as safe and admissible; and I came to the conclusion, that the mischief so generally dreaded or experienced, was justly attributable to the insufficiency of the dose used.

I was well aware that many articles of our Materia Medica could be made to produce opposite, or very different effects, by regulating the dose. Dr. Cartwright had very satisfactorily demonstrated that this principle applied to quinine; and long and careful observation had convinced me, that the principle was most peculiarly applicable to opium. Medical practitioners with whom I was thrown in contact questioned its utility in fever, on the ground that it aggravated the tendency to coma; and when induced by me to try the large dose, in cases where there was not only a tendency to coma, but where it actually existed in sufficient degree, as in typhoid fever, to destroy consciousness, they were surprised at the sudden and permanent relief obtained from a single dose; but so strong were their prejudices and their reverence for authority, that at first they would leave the patient with the conviction, that the remedy had been given just on the eve of a "crisis," and that he had recovered, "in spite of the opium," rather than as the result of its curative power. But I am happy to know, that those gentlemen are now warm advocates for the practice when an opiate is admissible; and are in the daily habit of giving from four to six grains, with less hesitation than they formerly felt when administering the one or two grain dose.

The large dose of opium is usually objected to on the ground that there is danger of producing fatal somnolency, especially where there is febrile excitement; but my experience is, that nothing is to be apprehended on that score, and that the large dose will not produce as much disposition to sleep as a dose of two grains.

In a paper publishsd in the Missouri Medical Journal, in the summer of 1845, I submitted the following propositions:

1st. That a four or six grain dose would produce less disposition to sleep, than one and a half or two grains.

2d. That ten grains of calomel, combined with four or six of opium, would produce a better alterative effect in fever, than thirty without the opium, provided they were both retained in the system the same length of time.

3d. That the large dose produces less constipation of the bowels than small doses.

Why this is so, it is not my purpose to inquire at this time. Those who question the truth of the propositions have only to try the experiment, to be convinced of their truth ; my object at preseut is to state the simple results of my observations and experience.

I regard opium, when used in the way I propose, as the most safe and efficient remedy in all forms of continued fever, to be found in the whole list of our remedial agents, calomel not excepted. I would not be understood as relying upon it exclusively, in the treatment of fever or any other form of disease ; but I do mean to say, that instead of relying upon calomel as my main remedy, as I was in the habit of doing in the outset of my professional career, I now regard it as an important auxiliary remedy, and opium as my "sheet anchor." I do not hesitate to affirm, that I can accomplish more in practice, in all forms of fever, by opium, quinine, &c., without mercury, than was ever secured by the old mercurial plan of treatment; and the difference in speediness of cure, and saving of human suffering is immense.

In lookidg over my journal of fever cases, treated on the mercurial plan, I find that the average number of days' attendance was about eight; and the mortality, one in twenty-seven. Under the opium plan of treatment, the average number of days' attendance has been about four; and the mortality not one in ninety. This estimate includes all the fever cases that came into my hands, in a general town and country practice; and it also includes all the various forms of fever, from the mildest bilious remittents, to the most malignant forms of congestive and typhus.

The following case will illustrate my mode of treatment, better than I can do it in any other way.

I was called on the evening of the 5th of September last, to see Mr. ———, aged about thirty-seven years, a carpenter by trade, who who had usually enjoyed robust health. He had been more or less indisposed for several days, but did not take his bed until the morning of the day I saw him. I found him with high fever, skin hot and dry, tongue heavily coated, red and glossy at the end, and complaining of violent pain in the head and back, distressing nausea, with tenderness over the stomach and bowels. He had been freely purged during the day, having taken a dose of Cooke's pills in the morning; consequently I gave him four grains of opium, and twelve grains of calomel, (without preceding the dose with ipecac and calomel, for the purpose of freely evacuating the stomach and bowels, as I am in the habit of doing,) and directed him to drink freely of hot sage tea. Found him on the the morning of the 6th, much improved in appearance; said he had rested well during the night; perspiring very freely. Fever was beginning to rise. I directed salts and senna to be taken during the day, until the bowels were freely moved, and left him the same dose to be taken at bed time. Found him on the morning of the 7th, entirely free from fever. The pain in the head and back had returned with fever, but with less intensity, but had entirely passed off in about an hour after he took the opium and calomel. The salts and senna had operated well the day before. Tongue still coated but moist. I directed five grains of quinine to be given at intervals of four hours, until three doses had been taken. I found him on the morning of the 8th, decidedly convalescent. Had no return of fever the day before, and expressed himself as feeling quite well. His tongue had cleaned off, and presented that peculiar tremulous motion indicating slight ptyalism; gums slightly swollen and tender to the touch. I directed a Seidlitz powder to be taken every hour, until the bowels were freely moved. Found him on the morning of the 9th, sitting up and doing well, with less

1*

indications of ptyalism, than the day before. In the course of three or four days he was at his work as usual.

I have selected this case because it was one of more than ordinary severity for a case of our endemic remittents; and also for the reason, that the cerebral affection constituted a prominent feature in the case. The result shows that determination to the head does not forbid the use of large doses of opium.

It may be urged that in this case, the cure was more attributable to the calomel than to the opium; but I am satisfied the fever would have continued without intermission together with the pain in the head and back for days longer, and perhaps have terminated fatally, if the opium had been omitted. To demonstrate this, I have in similar cases omitted the calomel with nearly the same results; the only difference being, that the patients did not get up so soon, and were more liable to relapse, but I did not fail to secure an intermission with two or three doses. But in my opinion, the calomel ought not to be dispensed with, except in cases where there is danger of profuse ptyalism with the most cautious use of it.

While I am satisfied that the large doses of opium can be used both with safety and efficiency in cases attended with cerebral affection, I am equally well convinced that they are entirely inadmissible where actual inflammation exists. We may have very serious *affection* of the brain and spinal chord, without inflammation. It may well be doubted whether we ever have inflammation without its being preceded by a stage of irritation, and hence the importance of allaying the irritation, more especially in fever, before inflammation is developed. In our typhoid forms of fever, I believe the affection of the brain is most generally the result of diminished power, and is only to be relieved by sedative and stimulant remedies. It may be that it is on this principle, that a large dose of opium and quinine so generally relieves the tendency to coma, and even coma itself, in this type of fever; or where too active depletion has been used, with a view of guarding the brain from injury.

I am not prepared to say what the specific action of a large opiate is; but I am well convinced that opium has an action altogether different from any that has been assigned to it by writers on Materia Medica. I am inclined to attribute its curative power in fever, partly to its sedative power, but mainly to its diaphoretic effects. I have been led to this conclusion by the fact, that no beneficial result is obtained in fever, until it is given in sufficient quantity to produce perspiration; and by the additional fact, that the benefit derived from its action is far greater than can be secured by any of our diaphoretic remedies, not excepting Dover's powder, which has opium for its basis. If we adopt Cullen's doctrine, of "spasm of the extreme vessels," for our theory of fever, we have a satisfactory explanation of the superior efficacy which I claim for the opium practice over all others that have been recommended.

In addition to the general satisfaction I derived, from the perusal of the very able and practical paper of Prof. Rogers, on "Carbonagogue Medication," recently published in this Journal, I feel particularly obliged to him for the satisfactory explanation he incidentally furnishes of the action of my remedy in fever. He says:

"Whatever hypothesis be entertained as to the etiology of fever, a prominent and pervading condition attending this affection, in its various forms, is an arrest or vitiation, of greater or less duration, and greater or less intensity, of the several most important secretory and excretory processes. This arrest or impairment is universally recognized as a potential part of the febrile combination; it constitutes a link in the chain of morbid phenomena, the severance of which generally resolves the difficulty. In what consists its importance? The physiology of these secretory and excretory processes explains at once the noxious influences of their pathological aberrations. *They are the scavengers of the system, and when they cease their labors, poisonous and polluting accumulations must occur.* Of these accumulations, the product of the natural waste of the body, *and possibly the poison of fever itself,* carbon constitutes an important, if not the chief element."

I have italicised a portion of the foregoing paragraph, by way of calling attention to the doctrine hinted at, as having a most important bearing upon the action of the remedy I propose, for meeting the great leading indication in fever.

It will be borne in mind that I have fixed the minimum and maximum dose, at four and six grains. In the generality of cases, four grains will fulfill the indication; but in cases where opium has been frequently resorted to by the patient as a household remedy, five or six grains will be required. I have given the largest dose hundreds of times, without having in any one instance, to my knowledge, done mischief by it. It may frequently occur, that patients will breathe rather heavily for an hour or two, but the perspiration will not fail to remove all unpleasant symptoms. Where the dose is given at night, there will be more or less nausea for an hour or two in the morning, a thing hardly worthy of notice, but it may be avoided entirely, by giving a single grain of opium eight or ten hours after the large dose, so as to let the system down gradually. I greatly prefer the solid opium to any of its preparations; even were it possible to determine the strength of the preparations of morphine, I would not use them in large doses, for I believe they have a different action from opium.

I cannot too strongly enforce upon those who may feel disposed to give the remedy a trial the great impropriety, if not unfairness, of delaying a resort to it, until they have exhausted all their favorite remedies. It may be given just as the case is on the eve of terminating in fatal coma from effusion, and the remedy would run the risk of being held responsible for an effect that depended entirely upon the disease. Let it be given early in the disease, and there will be nothing to be apprehended from coma, and comparatively little from a fatal termination.

In Inflammation, opium can only be used advantageously in the irritative, or forming stage.

I am indebted to the late Professor Barbour, of Saint Louis, (who adopted this plan of treating fever at my suggestion,) for a very valuable hint in regard to the use of opium in acute inflammation. He was in the habit, up to the time of his death, of administering the large dose before opening a vein in the arm, with a view of continuing the sedative effect of the bleeding. In this way, a single bleeding, with a large opiate, you will cure the most violent attacks of Pleurisy, Hepatitis, Gastro-Enteritis, Puerperal Fever, &c. In the latter most frightful disease, it will relieve every alarming symptom, and very often without a bleeding.

In the treatment of Dysentery, opium in large doses, may be regarded as approximating very nearly to a specific; for I am not aware of its ever having failed in my hands to arrest the disease, when resorted to at the onset of the attack, or before lesion had taken place in the bowel. The following case will show my mode of treating it:

In September last, I was called to see Mr. ———, a laborer, aged forty years. He had been attacked the evening before with violent griping of the bowels, distressing tenesmus, with frequent evacuations of bloody mucus, and high fever. I directed enemata of salt and water, which freely evacuated the bowels. I then administered five pills composed of one grain of opium, and three of calomel each, and directed him to drink freely of hot tea.

I called to see him two hours after, and found him entirely free from pain or uneasiness of the bowels, and perspiring freely. I left him three gr ins of opium, to be given in the event of a return of the dysenteric symptoms. Saw him twenty-four hours afterwards. He had remained quiet without taking the opium. I directed him to take a dose of oil, in the event of his bowels not being moved in the course of five or six hours. Called the next day and found him apparently well. His bowels had been moved the day before, without the aid of the oil. I directed him to remain quiet for a day or two, living on tea and toast, and discharged him cured. Two days after he was at his work, and had no return of the disease.

After evacuating the bowels with a saline cathartic or enema, I aim to give a dose large enough to quiet the bowels at once, and I regard it as very important, that they should be kept quiet for twenty-four, or thirty-six hours, or until all disposition to a return of the dysenteric symptoms has subsided. If you give a cathartic at the end of ten or twelve hours, (as some of my medical brethren here, who have adopted the large opiate, are in the habit of doing,) you are almost certain to re-produce the disease, and involve the necessity of starting anew with your treatment. I regard this as a very great error, and one that frequently proves fatal. The principal reason urged in favor of the practice is, that you incur the risk of a sore mouth, if you withhold the cathartic. But I insist that you had better risk a sore mouth, than the life of your patient, or at best, days of unnecessary suffering. There is however, no necessity for incurring the risk of either. If your patient is very impressible to calomel, give less, or omit it altogether. I prefer giving it with the opium, not only for its action upon the liver, but for its cathartic effect after the influence of the opium has passed off. Still, I am fully satisfied you can cure the disease with opium, without the aid of mercury in any form.

The opium plan of treatment, will be found quite as efficacious in all our typhoid forms of fever, as in bilious remittents. When resorted to in the commencement of the disease, it will seldom fail to arrest its progress, or place it entirely within your control. In that most malignant form of fever, congestive typhus, where the brain seems to be overwhelmed by the force of the poison, at the very onset of the attack, a large opiate combined with quinine, is the only remedy in my opinion, entitled to confidence. Bleeding and other evacuant remedies, so far as my observation extends, fail to give relief, and the loss of time places the patient beyond the reach of all remedy.

I trust I have succeeded in making myself clearly understood; and I indulge the hope, that my readers will give the practice a fair trial, and report the results of their experience to the profession.

I deem it due to myself to assert my claim to the priority of whatever may be valuable in this new mode of using opium. I do this now, in consequence of having noticed lately in the medical journals allusions to the use of large doses of opium in fever, &c., without the slightest reference to myself as the originator of the practice; and for the more important reason, that Prof. Drake in his reference to the practice in his valuable work recently published, gives the credit of the practice to "Merryman and Henry." Dr. Drake was naturally led into the commission of this error by the circumstance that I was connected with Dr. Merryman in the practice at the time of Dr. Drake's visit to Springfield in 1840.

November, 1851.

